# BJ-1108, a 6-Amino-2,4,5-trimethylpyridin-3-ol analogue, regulates differentiation of Th1 and Th17 cells to ameliorate experimental autoimmune encephalomyelitis

**DOI:** 10.1186/s40659-017-0113-z

**Published:** 2017-02-28

**Authors:** Youra Kang, Maheshwor Timilshina, Tae-gyu Nam, Byeong-Seon Jeong, Jae-Hoon Chang

**Affiliations:** 1 College of Pharmacy, Yeungnam University, Gyeongsan, 38541 Republic of Korea; 2 Department of Pharmacy and Institute of Pharmaceutical Science and Technology, Hanyang University, Ansan, Gyeonggi-do 15588 Republic of Korea

**Keywords:** BJ-1108, Th1/Th17 cell, Differentiation, EAE

## Abstract

**Background:**

CD4^+^ T cells play an important role in the initiation of an immune response by providing help to other cells. Among the helper T subsets, interferon-γ (IFN-γ)-secreting T helper 1 (Th1) and IL-17-secreting T helper 17 (Th17) cells are indispensable for clearance of intracellular as well as extracellular pathogens. However, Th1 and Th17 cells are also associated with pathogenesis and contribute to the progression of multiple inflammatory conditions and autoimmune diseases.

**Results:**

In the current study, we found that BJ-1108, a 6-aminopyridin-3-ol analogue, significantly inhibited Th1 and Th17 differentiation in vitro in a concentration-dependent manner, with no effect on proliferation or apoptosis of activated T cells. Moreover, BJ-1108 inhibited differentiation of Th1 and Th17 cells in ovalbumin (OVA)-specific OT II mice. A complete Freund’s adjuvant (CFA)/OVA-induced inflammatory model revealed that BJ-1108 can reduce generation of proinflammatory Th1 and Th17 cells. Furthermore, in vivo studies showed that BJ-1108 delayed onset of disease and suppressed experimental autoimmune encephalomyelitis (EAE) disease progression by inhibiting differentiation of Th1 and Th17 cells.

**Conclusions:**

BJ-1108 treatment ameliorates inflammation and EAE by inhibiting Th1 and Th17 cells differentiation. Our findings suggest that BJ-1108 is a promising novel therapeutic agent for the treatment of inflammation and autoimmune disease.

## Background

CD4^+^ T cells play an important role in adaptive immunity by orchestrating other immune cells [1]. Upon antigenic exposure, naïve CD4^+^ T cells undergo differentiation and expansion of distinct effector subsets, which play a major role in mediating immune responses through the secretion of specific cytokines [2, 3]. The differentiation of naïve CD4^+^ T cells begins with antigenic stimulation, which results in interactions between the T cell receptor (TCR), with CD4 as a co-receptor, and the antigen-MHC II complex presented by antigen presenting cells (APCs) [3]. TCR signaling induces downstream signaling that leads to proliferation and differentiation of naïve CD4 T cells into effector cells [4]. Lineage-specific differentiation depends upon TCR signaling, the cytokine environment, and co-stimulatory molecules that direct differentiation of naïve CD4^+^ T cells into IFN-γ-secreting T-helper 1 (Th1), IL-4-secreting T-helper 2 (Th2), IL-17-secreting T-helper 17 (Th17), and IL-10-secreting regulatory T (Treg) cells [1, 5]. Th1 cells participate in the elimination of intracellular pathogens and regulation of organ-specific autoimmune diseases [1]. Similarly, Th17 cells enhance immune responses against extracellular pathogens, particularly bacteria and fungi, as well as tissue inflammation [2, 6]. Nevertheless, unrestrained activation of Th1 and Th17 cells is associated with autoimmune and inflammatory disorders such as multiple sclerosis, rheumatoid arthritis, and psoriasis [7, 8].

Autoimmune diseases are abnormal immune responses in which activation and expansion of autoreactive T cells and other inflammatory cells play important roles in tissue inflammation and injury [9, 10]. Multiple sclerosis (MS) is one of the most common autoimmune diseases of the central nervous system. In MS, inflammatory cells infiltrate and demyelinate the axonal tract in the brain and spinal cord, disrupting neuronal signaling along axons [11]. Finally, neurodegeneration of the brain and spinal cord, mediated by CD4^+^ T cells directed against myelin, can result in paralysis [12]. Experimental autoimmune encephalomyelitis (EAE) is an animal model of MS that mimics the clinical and pathophysiological features of human MS [13, 14]. Although the exact cause of MS is unclear, it is thought to be mediated by a combination of genetic and environmental factors [10, 15–17]. Although Th1 cells are considered to be the primary effector T cells in EAE pathology, EAE can occur in IFN-γ knockout mice [18]. Previous studies have shown that Th17 cells that secrete IL-17 and IL-23 are also important to the development of EAE [19–21]. Altogether, the studies provide evidence that both proinflammatory Th1 and Th17 cells are associated with pathogenesis of autoimmune diseases like multiple sclerosis and rheumatoid arthritis [22, 23]. MS affects more than 2 million people worldwide. A number of chemotherapeutic and immunotherapeutic agents have been approved as MS disease-modifying therapies [24–27]. However, these therapies are associated with serious side effects and frequent response failures, and safe medications to manage autoimmune and inflammatory diseases are still needed.

Previous studies have shown that BJ-1108, an analogue with a phenyl group attached to a 6-amino moiety strongly inhibits angiogenesis and tumor growth [28, 29]. Inflammation is one of the major pathophysiological characteristics of autoimmune disease and is associated with oxidative stress and reduction in cellular antioxidant capacity [30]. 6-Amino-2,4,5-trimethylpyridin-3-ol analogues have been reported to show antioxidant and antiangiogenic activity [31, 32]. Furthermore, Timilshina et al. reported that a 2,4,5-trimethylpyridin derivative inhibits Th1 and Th17 differentiation and subsequently ameliorates EAE progression [33]. These findings prompted us to examine whether BJ-1108 could be used to treat an inflammatory autoimmune disease like MS, using an EAE model.

We investigated the therapeutic potential of a novel derivative (6-amino-2,4,5-trimethylpyridin-3-ol; BJ-1108) on inflammation and autoimmune disease. We found that BJ-1108 significantly suppressed Th cell function by inhibiting Th1 and Th17 differentiation and marginally decreased proliferation of activated T cells without apoptosis. Further, we found that BJ-1108 treatment reduced Th1 and Th17 generation in a complete Freund’s adjuvant (CFA)/OVA-immunized inflammatory model. Furthermore, BJ-1108 treatment delayed the onset of EAE and alleviated ongoing EAE by reducing infiltration of mononuclear cells into the central nervous system (CNS), as well as decreased Th1 and Th17 cells in the spleen, draining lymph nodes (dLNs), and CNS of EAE-affected mice.

## Results

### BJ-1108 inhibits differentiation of Th1 and Th17 cells

**Fig. 1:**
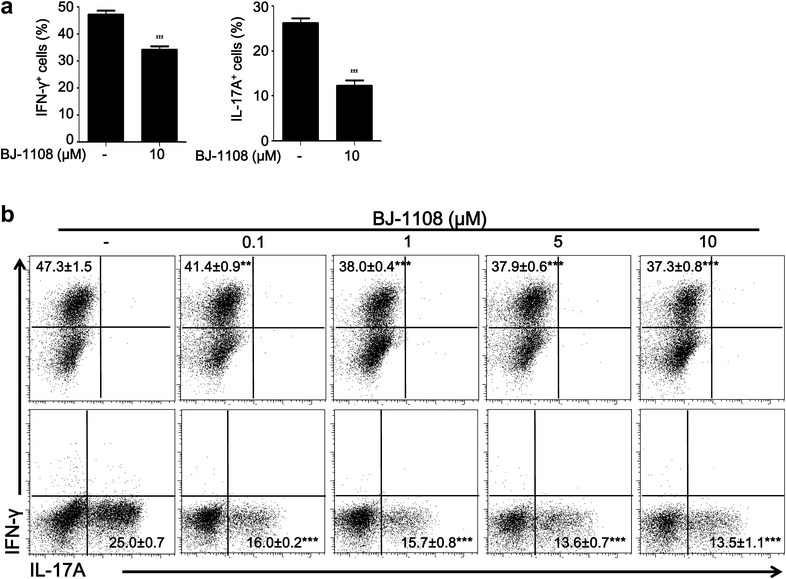
BJ-1108 inhibits CD4^+^ T cell differentiation. **a** Naïve CD4^+^ T cells isolated from spleens and draining lymph nodes were stimulated under Th1- and Th17-polarizing conditions in the presence or absence of 10 μM BJ-1108 for 72 h. Cells were then re-stimulated with phorbol 12-myristate 13-acetate, ionomycin, and GolgiStop for 4 h, followed by intracellular cytokine staining and flow cytometry. **b** Th1 and Th17 differentiation with multiple concentrations of BJ-1108. Representative data (mean ± SEM) of three independent experiments are shown. **p < 0.001 and ***p < 0.0001 versus vehicle

Based on reports that 6-aminopyridin-3-ol analogues inhibit oxidative stress and inflammation [29], we examined whether BJ-1108 is involved in autoimmunity and inflammatory immune responses. CD4^+^ T cells are essential to an immune response, and Th1 and Th17 cells have been extensively studied to understand inflammation and autoimmune diseases [34, 35]. Inhibiting differentiation of naïve CD4^+^ T cells into proinflammatory Th1 and Th17 cells helps to mitigate autoimmune disease [36]. To test the inhibitory effect of BJ-1108 on Th1 and Th17 differentiation, purified splenic CD4^+^ T cells were cultured in Th1 and Th17-polarizing conditions with cytokine stimulation and TCR ligation by anti-CD3 and anti-CD28 for 3 days. Under Th1-polarizing conditions, approximately 54% of CD4^+^ T cells were IFN-γ^+^ in the untreated control group, and BJ-1108 treatment significantly inhibited Th1 differentiation by as much as 37%. In addition, up to a 50% reduction group in Th17 differentiation was observed in the BJ-1108-treated mice. Therefore, BJ-1108 (10 μM) treatment significantly reduced IFN-γ^+^ and IL-17^+^ cells differentiation on day 3 after in vitro stimulation with TCR and cytokines (Fig. 1a). To further investigate the regulatory effects of BJ-1108 on CD4^+^ T cells differentiation, CD4^+^ T cells stimulated by TCR and cytokines were treated with varying concentrations of BJ-1108. BJ-1108 treatment decreased the percentage of IFN-γ^+^ Th1 and IL-17^+^ Th17 cells in a concentration-dependent manner (Fig. 1b). These data suggest that BJ-1108 significantly decreased differentiation of Th1 and Th17 cells.

### BJ-1108 inhibits antigen-specific CD4^+^ T cell differentiation

**Fig. 2:**
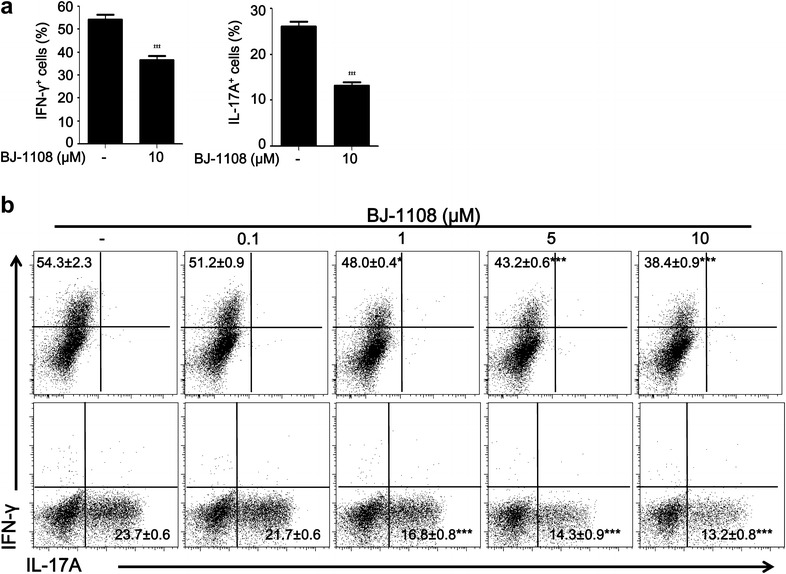
BJ1108 negatively regulates CD4^+^ T cells differentiation without antigen specificity. Naïve CD4^+^ T cells and antigen presenting cells isolated from spleen and LNs of OT-II mice. Cells were cultured under Th1- and Th17-polarizing conditions with OVA_323–339_ (0.1 μM) in the presence or absence of a **a** single concentrations (10 μM) or **b** multiple concentrations of BJ-1108. Cells were then re-stimulated with phorbol 12-myristate 13-acetate, ionomycin, and GolgiStop for 4 h, followed by intracellular cytokine staining and flow cytometry. Representative data (mean ± SEM) of three independent experiments are shown. *p < 0.01 and ***p < 0.0001 versus vehicle

To examine whether BJ-1108 can inhibit antigen-specific Th1 and Th17 differentiation of CD4^+^ T cells, we used ovalbumin (OVA)-specific OT-II TCR transgenic mice. OT-II CD4^+^ T cells express transgenic alpha-chain and beta-chain TCRs that are specific for chicken OVA 323–339 in the context of MHC class II [37]. Naïve CD4^+^ T cells were isolated from spleens and lymph nodes (LNs) of OT-II TCR transgenic mice and cultured with BJ-1108 in the presence of OVA peptide and APCs for 3 days. Consistent with Fig. 1a, BJ-1108 inhibited generation of IFN-γ^+^ CD4^+^ T cells by 30% and IL-17^+^ CD4^+^ T cells by 50% (Fig. 2a). To examine the effects of BJ-1108 on OVA-specific Th1 and Th17 differentiation, OT-II CD4^+^ T cells were treated with various concentrations of BJ-1108 in the presence of OVA peptide and APCs. The percentage of IFN-γ-producing Th1- and IL-17-producing Th17 cells was decreased in a concentration-dependent manner by BJ-1108 (Fig. 2b). Generation of IL-17-secreting Th17 cells was suppressed more than IFN-γ-secreting Th1 cells by treatment with BJ-1108. Thus, BJ-1108 can directly inhibit differentiation of antigen-specific T cells.

### BJ-1108 has no significant effect on T cell proliferation

**Fig. 3:**
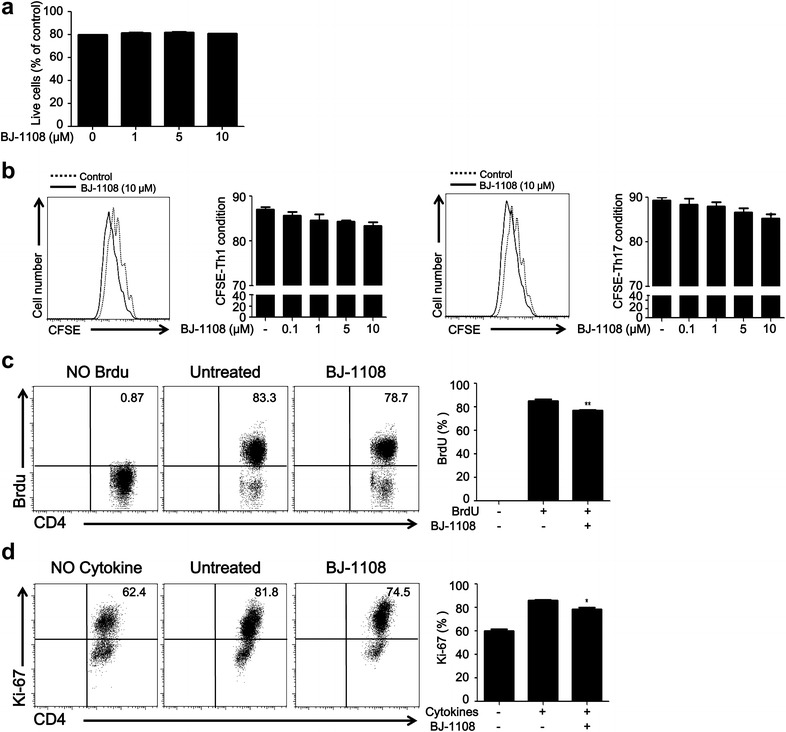
BJ-1108 partially inhibits CD4^+^ T cell proliferation without apoptosis. **a** Naïve CD CD4^+^ T cells and antigen-presenting cells (APCs) isolated from spleen and lymph nodes (LNs). Apoptosis was analyzed by Annexin-V and propidium iodide staining after cells were cultured in Th1-differentiating conditions for 72 h, followed by flow cytometry. The percentages of live cells are shown. **b** Naïve CD4^+^ T cells and APCs isolated from spleen and LNs. CFSE-labeled naïve CD4^+^ T cells were cultured under Th1- (*left panel*) and Th17- (*right panel*) polarizing conditions. Histogram shows cell proliferation analyzed by CFSE dilution using flow cytometry.* Bar graphs* indicate the percentage of CFSE^+^CD4^+^ T cells. **c** Naïve CD4^+^ T cells and APCs isolated from spleen and LNs were cultured under Th1-polarizing conditions with BrdU (10 μM) in the presence or absence BJ-1108 (10 μM) for 72 h. Cells were analyzed by flow cytometry.* Bar graphs* indicate the percentage of BrdU^+^CD4^+^ T cells. **d** Naïve CD4^+^ T cells and APCs isolated from spleen and LNs were cultured under Th1-polarizing conditions in the presence or absence BJ-1108 (10 μM) for 72 h.* Bar graphs* indicate the percentage of Ki-67^+^CD4^+^ T cells. Representative data from three independent experiments are shown. *p < 0.01 and **p < 0.001 versus untreated group

To test whether the regulatory effect of BJ-1108 on Th cell differentiation is mediated by cytotoxicity or reduced proliferation, we checked the effect of our compound on apoptosis and proliferation of CD4^+^ T cells. CD4^+^ T cells were isolated and cultured under anti-CD3 and anti-CD28 stimulation in the presence or absence of BJ-1108 for 3 days. On day 3 after activation, apoptosis was assessed with Annexin-V and propidium iodide (PI) staining. The percentages of viable cells were comparable between untreated cells and those treated with various concentrations of BJ-1108 (Fig. 3a). Next, carboxyfluorescein succinimidyl ester (CFSE)-labeled CD4^+^ T cells were cultured with various concentrations of BJ-1108 in Th1- and Th17-polarizing conditions for 3 days. Based on CFSE dilution, treatment with different concentrations of BJ-1108 demonstrated a slight decrease in Th1 and Th17 cell proliferation (Fig. 3b). However, decrease in proliferation was negligible compared to BJ-1108-mediated differentiation. Furthermore, in vitro proliferation measured by thymidine analog bromodeoxyuridine (BrdU) labeling assay demonstrated that BJ-1108 treatment slightly decreased proliferation under Th1-polarizing conditions (Fig. 3c). Similarly, Ki-67, a nuclear protein indicating cell proliferation, was analyzed after 3 days of culture under Th1-polarizing conditions. Proliferation of IL-12-treated cells increased in a manner relative to that of cells not treated with cytokine, whereas BJ-1108 treatment reduced the rate of Ki-67 expression to less than 10% of that in cells not treated with the compound (Fig. 3d). Altogether, these data suggest that although BJ-1108 slightly affects CD4^+^ T cell proliferation, but that inhibition of Th cell differentiation is not a result of reduced proliferation or increased apoptosis.

### BJ-1108 reduces the inflammatory response in CFA/OVA-immunized mice

**Fig. 4:**
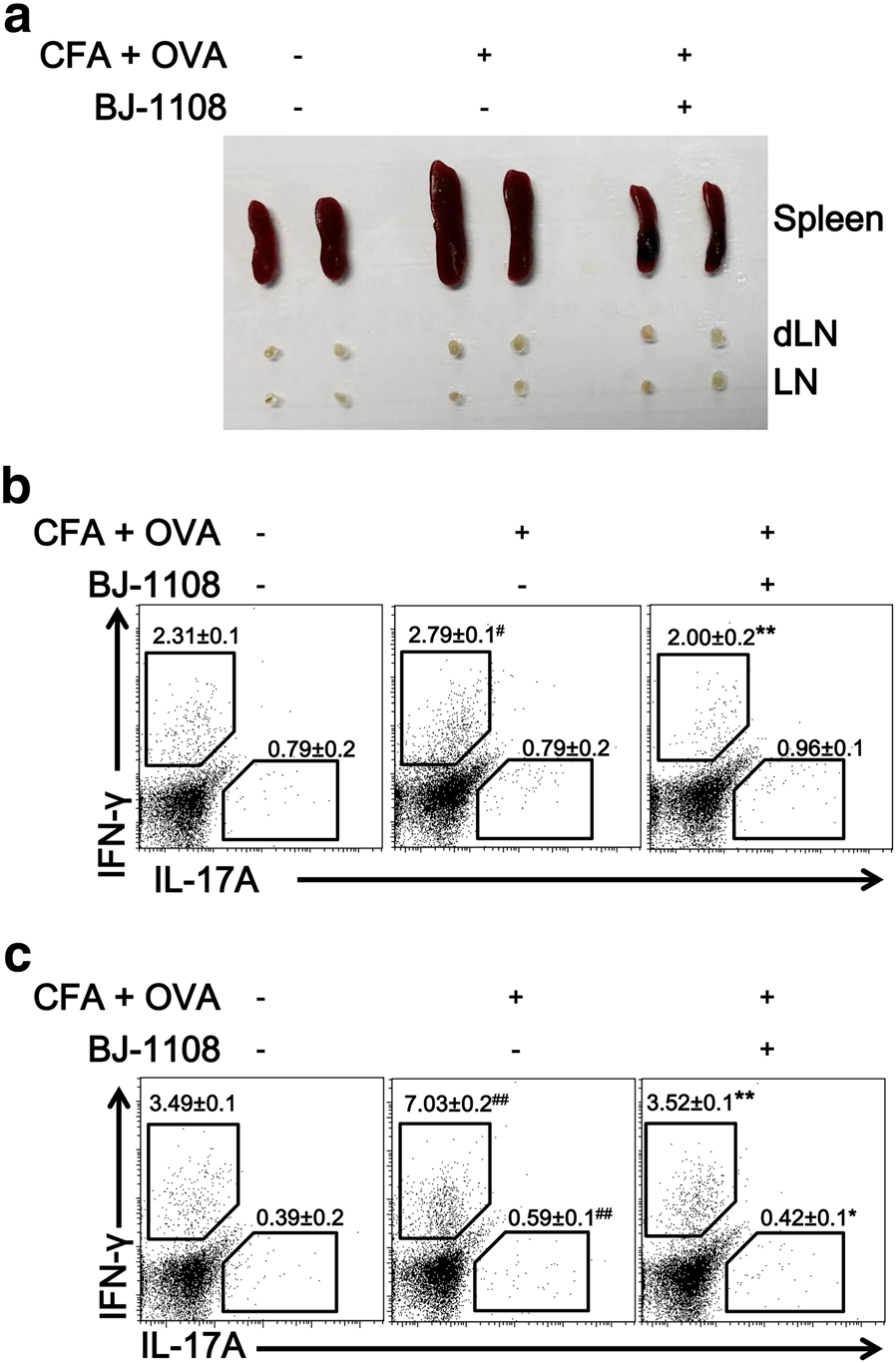
Suppression of inflammation in vivo by BJ-1080 in complete Freund’s adjuvant/ovalbumin (CFA/OVA)-immunized mice. Acute inflammation was induced in 8- to 12-week-old C57BL/6 mice by intraperitoneal immunization with OVA in CFA, and then 1× PBS or 1 mg/kg BJ-1108 was administered intraperitoneally each day. **a** Images of spleens, lymph nodes, and draining lymph nodes (dLNs) from CFA/OVA-immunized mice treated or untreated with BJ-1108 after 4 days. CD4^+^ T cells from **b** dLNs and **c** spleens were re-stimulated with phorbol 12-myristate 13-acetate and ionomycin for 4 h, followed by measurement of IFN-γ- and IL-17A-producing CD4^+^ T cells by flow cytometry. Numbers in the dot plots represent percentages of Th1 and Th17 cells. The mean ± SEM of five independent experiments is shown. ^*#*^ *p* < 0.01 versus vehicle. *p < 0.01 and **p < 0.001 versus CFA/OVA-treated group

Th1 and Th17 cells are critical for the progression and pathology of inflammation and autoimmune diseases [8]. Inhibition of Th1 and Th17 cell differentiation in vitro by BJ-1108 prompted us to examine whether this compound could inhibit inflammatory responses initiated by IFN-γ and IL-17A. Mice were administrated OVA (2 mg/ml) in CFA by intraperitoneal injection. CFA/OVA administration induced inflammation through the generation of Th1 and Th17 cells. BJ-1108 (1 mg/kg) was injected every day for up to 4 days, and mice were sacrificed on day 5. We found that the size of spleens, lymph node (LN) and draining lymph nodes (dLNs) in BJ-1108-treated CFA/OVA-immunized mice were smaller than those in mice immunized with CFA/OVA alone (Fig. 4a). Furthermore, Th cells from spleens and LNs of CFA/OVA-immunized mice that received either BJ-1108 or no treatment were analyzed. The results showed that CFA/OVA administration promoted IFN-γ and IL-17A generation as compared to no CFA/OVA immunized mice, and BJ-1108 treatment inhibited generation of IFN-γ and IL-17A in LNs and spleens in CFA/OVA immunized mice (Fig. 4b, c). Thus, BJ-1108 inhibits inflammation by reducing IFN-γ-producing Th1 and IL-17A-producing Th17 cells in vivo.

### BJ-1108 attenuates EAE pathology by negatively regulating inflammatory T cells

**Fig. 5:**
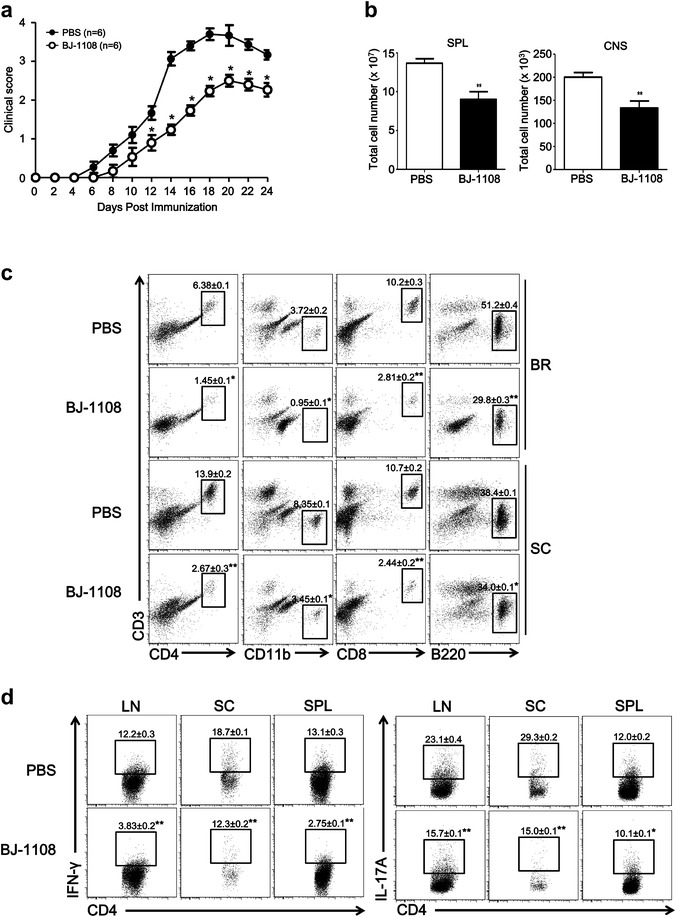
BJ-1108 ameliorates the onset and development of experimental autoimmune encephalomyelitis (EAE) by attenuating the generation of Th1 and Th17 cells. Acute EAE was induced in 8- to 12-week-old C57BL/6 mice by subcutaneous immunization with MOG_35–55_ in complete Freund’s adjuvant and pertussis toxin. Mice were administered 1 mg/kg BJ-1108 or vehicle intraperitoneally each day. **a** Clinical scores were assigned daily. **b** Total cell count in spleen and CNS of drug treated and untreated EAE mice. **c** Twenty-four days later, total mononuclear cells were isolated from brains and spinal cords of mice and analyzed by flow cytometry. Total percent of infiltrated CD4^+^ T cells, CD8^+^ T cells CD11^+^ cells and B220^+^ cells in CNS. **d** 24 days later, lymphocytes from spleen, LNs, and spinal cords were re-stimulated with phorbol 12-myristate 13-acetate and ionomycin for 4 h, followed by measurement of IFN-γ- and IL-17A-producing CD4^+^ T cells using flow cytometry.* Numbers* in the* dot plots* represent percentages of Th1 and Th17 cells. The mean ± SEM of five independent experiments is shown. *p < 0.01 and **p < 0.001 versus vehicle

The finding that BJ-1108 inhibited Th1 and Th17 differentiation in vitro and reduced inflammation by decreasing IFN-γ-producing Th1 and IL-17A-producing Th17 cells in vivo prompted us to investigate whether BJ-1108 treatment affects the development of inflammatory autoimmune disease. To address this question, we employed the EAE model, a well-established model of MS, because Th1 and Th17 cells are critical for the progression and pathology of MS [21]. To investigate the possible protective role of BJ-1108 in the development of EAE, we immunized female C57BL/6 mice with MOG_35−55_ peptide emulsified with CFA and pertussis toxin as described in “Methods” section. Vehicle or BJ-1108 (1 mg/kg) was administrated intraperitoneally every other day beginning 1 day after immunization. The severity of the resulting paralysis was assigned a disease score. All mice in the vehicle-treated group developed severe EAE with a mean peak clinical score of 3.5, whereas BJ-1108-treated mice showed delayed disease onset and significantly diminished EAE severity, with a 2.6 mean peak clinical score (Fig. 5a). The total cell number from spleen and CNS were also decreased in drug treated EAE mice (Fig. 5b). Furthermore, CNS-infiltrated mononuclear cells were enriched by density gradient centrifugation and analyzed by flow cytometry. As depicted in Fig. 5c, significantly reduced infiltration of CD4^+^ T cells, CD8^+^ T cells, B220^+^ B cells, and CD11b^+^ macrophages/microglia was observed in the brains and spinal cords of BJ-1108-treated EAE mice. Because autoreactive CD4^+^ T cells, especially Th1 and Th17 cells, are critical to the induction of EAE, we analyzed Th cells in EAE mice. As expected, BJ-1108 treatment significantly reduced IFN-γ-secreting Th1 and IL-17-secreting Th17 cells in spleens, dLNs, and CNS of EAE-induced mice (Fig. 5d). These data suggest that BJ-1108 is effective in ameliorating ongoing EAE by restricting Th1 and Th17 cell differentiation.

## Discussion

Our study demonstrated BJ-1108 suppression of Th1 and Th17 cell differentiation with no effect on proliferation and apoptosis of activated T cells in vitro. BJ-1108 restricted CFA/OVA-induced inflammation by reducing IFN-γ-producing Th1 and IL-17A-producing Th17 cells in vivo. Furthermore, BJ-1108 treatment alleviated inflammatory infiltration and reduced leakage of mononuclear cells from the blood brain barrier. Mice that received BJ-1108 treatment displayed lower EAE scores and better clinical recovery from EAE. Moreover, BJ-1108 administration reduced the frequencies of Th1 and Th17 cells in the spleens, LNs, and spinal cords of EAE mice.

CD4^+^ Th cells play an important role in activating and directing other immune cells [1]. IFN-γ secretion-induced Th1 cell differentiation depends on signaling through IFN-γ receptor, IL-12 receptor and their downstream signaling transcription factor signal transducer and activator of transcription 1 (STAT1) and STAT4. Likewise, IL-17-producing Th17 cell differentiation is initiated after IL-6 stimulation and subsequent activation of STAT3 [36]. These proinflammatory Th1 and Th17 cells are key mediators of inflammation and the development of autoimmune disease. Th1- and Th17-associated cytokines have a significant impact on inflammation in the brain and severity of disease [38, 39]. The attenuation of inflammation in BJ-1108-treated mice was associated with a decrease in the differentiation of Th1 and Th17 cells and therefore a reduction in IFN-γ and IL-17 cytokine expression in spleens, lymph nodes and CNS.

CD4^+^ T cell responses to antigen are instructed by innate immune factors. The environment in which APCs initially encounter antigens is associated with specific adjuvants. Presentation of processed antigen with co-stimulatory molecules and a precise combination of cytokines drives differentiation of naïve CD4^+^ T cells toward a specific effector lineage, including that of Th1, Th2 and Th17 cells [40]. Therefore, we used an OVA-based mouse inflammatory disease model in which OVA combined with CFA, a potent Th1/Th17 skewing adjuvant, induced a powerful Ova-specific Th1 and Th17 inflammatory immune response. BJ-1108 treatment inhibited inflammation of CFA/OVA-induced mice by negatively regulating differentiation of IFN-γ^+^ Th1 and IL-17^+^ Th17 cells.

EAE, an animal model of human MS, is mediated by autoreactive T cells that secrete pro-inflammatory cytokines in the CNS, leading to inflammation and demyelination [11, 12, 41]. Th1 cells have been considered the primary effector T cells in the pathology of EAE and MS [8, 42, 43]. However, accumulating evidence reveals that both Th1 and Th17 cells are crucial for autoimmune disease [8, 22, 44, 45]. Proinflammatory cytokines such as IFN-γ and IL-17, secreted by Th1 and Th17 cells, cause inflammation, and are primary causes for aggravation autoimmune disorder [44]. Therefore, investigating drugs that target Th1 and Th17 cells to manage autoimmune diseases has clinical significance. We provide in vitro and in vivo evidence that BJ-1108 represses the development of Th1 and Th17 cells and ameliorates EAE. BJ-1108 treatment significantly reduced the generation of Th1 and Th17 cells in spleens, dLNs, and CNS of EAE mice at the peak of disease. However, APCs such as microglia, astrocytes, macrophages, and B cells act as the first line of defense against infection or inflammation and can participate in self-destructive mechanisms by secreting inflammatory factors and/or presenting myelin epitopes to autoreactive T cells [46]. How BJ-1108 affects myeloid cell function is unknown; however, a significant reduction in infiltrating CD11b^+^ macrophages/microglia and B220^+^ B cells in the brain and spinal cord suggests that BJ-1108 may regulate myeloid cells by regulating T cell function.

The antioxidant effects of 6-amino-2,4,5-trimethylpyridin-3-ol scaffold have been reported in several studies [31, 32]. Recently, BJ-1108 was shown to significantly inhibit angiogenesis and reactive oxygen species (ROS) production in cancer cells [29]. T cells, especially Th1 and Th17 cells, function in tumor immunity by secreting cytokines and transcription factors [47]. ROS produced in response to NOX-2 are associated with the differentiation of T cells, but are not required for T cell activation or proliferation [48]. The current study revealed anti-inflammatory activities of BJ-1108 in an inflammatory disease model, mediated by a reduction in Th1 and Th17 cell differentiation. NOX-2-derived ROS are associated with T cells differentiation, but do not affect T cell proliferation and activation [48–50]. Bonini et al. reported that administration of ROS scavengers reduced EAE lethality in negative regulator of ROS (NRROS)-knockout mice [51]. NRROS interacts with NOX-2 and maintains its stability [51]. BJ-1108 significantly inhibits NOX-2-derived ROS, which may lead to reduced Th1 and Th17 differentiation [29]. Altogether, the studies suggest that the effects of BJ-1108 on T cell differentiation correlate with inhibition of NOX-2-derived ROS and subsequently ameliorate inflammation and autoimmune disease.

In conclusion, the current study revealed the therapeutic potential of BJ-1108 for inflammation and autoimmune diseases. BJ-1108 treatment reduced the severity of inflammation and EAE disease by inhibiting differentiation of naïve CD4^+^ T cells into Th1 and Th17 cells. However, because previous studies have indicated that Th1 and Th17 differentiation is caused by inhibition of NOX-2-derived ROS, further research is needed to define the precise target of BJ-1108. Collectively, these data imply that BJ-1108 could be a promising therapeutic compound for the management of Th1- and Th17-mediated inflammation and autoimmune disease.

## Methods

### Mice

C57BL/6 mice were maintained in pathogen-free conditions at the Animal Center of Yeungnam University. The gradual fill method of CO_2_ inhalation was used to euthanize mice with minimal pain. No animals died during the study. Animal experiments were approved by Institutional Animal Care and Use Committee (IACUC) of Yeungnam University (Approval No: 2015-029).

### Intracellular cytokine staining and flow cytometry

CD4^+^ T cells were collected and re-stimulated for 4 h with phorbol 12-myristate 13-acetate (PMA) (50 ng/ml; Sigma) and ionomycin (750 ng/ml; Calbiochem, La Jolla, CA, USA) with GolgiStop (BD Biosciences). Cells were stained with anti-mouse CD4-FITC (GK1.5; BioLegend, San Diego, CA, USA), anti-mouse B220-PE/Cy7 (RA3-6B2; BioLegend), anti-mouse CD3 ε-APC (145-2C11; BioLegend), anti-mouse CD8a-PE/Cy7 (53-6.7; BioLegend), anti-mouse IFN- γ-PE (XMG1.2; BioLegend), and anti-mouse IL-17A-APC (TC11-18H10.1; BioLegend) according to the manufacturer’s instructions. Data were obtained with a FACSVerse (BD Immunocytometry System, San Jose, CA, USA) and analyzed using FlowJo software.

### In vitro T cell differentiation assay

Naïve CD4^+^ T cells were positively selected from spleens and LNs using anti-CD4 microbeads (Miltenyi Biotec, Auburn, CA, USA). CD8^+^ cells were depleted using anti-CD8 microbeads (Miltenyi Biotec), and the remaining cells were regarded as APCs. CD4^+^ T cells and APCs were cultured in complete RPMI 1640 medium containing 10% fetal bovine serum (FBS) and 1% penicillin and streptomycin. For antigen-specific stimulation, isolated naïve CD4^+^ T cells (2 × 10^5^) and APCs ((1 × 10^5^) from OT-II mice were incubated with OVA_323–339_ peptide (0.1 μM) under Th1-polarizing conditions (10 ng/ml IL-12; BioLegend, 5 μg/ml anti-IL-4; BioLegend), Th17-polarizing conditions (1 ng/ml TGF-β1; R&D Systems, 10 ng/ml IL-6; R&D Systems, 5 μg/ml anti-IL-4; BioLegend, 5μ/ml anti–IFN-γ; BioLegend).

### T cell proliferation assays

Naïve CD4^+^ T cells were purified using microbeads (Miltenyi Biotec), followed by labeling with CFSE (eBioscience) in a 37 °C water bath for 15 min. CFSE-labeled naïve CD4^+^ T cells were stimulated with anti-CD3 (5 μg/ml) and anti-CD-28 (1 μg/ml) antibodies in Th1- and Th17-polarizing conditions. After 3 days, cell proliferation was measured with CFSE dye dilutions using flow cytometry. For 5-bromo-2′-deoxyuridine (BrdU) labeling, naïve CD4^+^ T cells from spleens and LNs were cultured under Th1-polarizing conditions with BrdU (10 μM). After 3 days, cells were stained using a BrdU kit according to the manufacturer’s protocol (BD Biosciences). For Ki-67 detection, naïve CD4^+^ T cells were cultured under Th1-polarizing conditions and stained with phycoerythrin-conjugated Ki-67 (BioLegend). BrdU and Ki-67 were measured using flow cytometry.

### Apoptosis assay

Naïve CD4^+^ T cells were purified using microbeads (Miltenyi Biotec) and cultured under Th1-polarizing conditions with anti-CD3 (5 μg/ml) stimulation. After 3 days, apoptosis was assessed by staining for Annexin V-APC and PI according to the manufacturer’s protocol (BD Biosciences), followed by flow cytometry.

### Immunization

To induce an inflammatory response, 6 to 8-week-old mice were immunized intraperitoneally with 2 mg/ml OVA and an equal volume of CFA in the presence or absence of 1 mg/kg BJ-1108 daily. After 5 days, spleens and dLNs were collected and analyzed by flow cytometry. To induce EAE, 6 to 8-week-old mice were immunized subcutaneously with 6 mg/ml MOG_35–55_ peptide (MEVGWYRSPFSRVVHLYRNGK) emulsified in CFA containing 5 mg/ml *Mycobacterium tuberculosis* H37RA (Difco). Mice were injected intraperitoneally with 250 ng pertussis toxin (List Biological Laboratories) on the day of immunization and 48 h later. Mice were monitored daily, and disease was scored as follows: 0 = normal; 1 = limp tail; 2 = paraparesis (limp tail and incomplete paralysis of one or two hind limbs); 3 = paraplegia (limp tail and complete paralysis of two hind limbs); 4 = paraplegia with forelimb weakness or paralysis; 5 = moribund appearance or death. One milligram per kilogram BJ-1108 in phosphate-buffered saline (PBS) or PBS only (vehicle) were administered intraperitoneally on day 0 and every other day subsequently.

### Statistical analysis

Data are expressed as the mean ± SEM. Student’s *t* test or one-way ANOVA were used to assess the significance of differences between experimental groups using Prism software (GraphPad).

## Abbreviations

IFN-γ: interferon-γ
Th1: T-helper 1
Th17: T-helper 17
MHC-II: major histocompatibility complex class II
TCR: T cell receptor
EAE: experimental autoimmune encephalomyelitis
S: multiple sclerosis
OVA: ovalbumin
APC: antigen presenting cell
MOG: myelin oligodendrocyte glycoprotein
LN: lymph node
dLN: draining lymph node
PMA: phorbol 12-myristate 13-acetate
CFSE: carboxyfluorescein succinimidyl ester
